# Mitochondrial sequence variants: testing imputation accuracy and their association with dairy cattle milk traits

**DOI:** 10.1186/s12711-024-00931-5

**Published:** 2024-09-12

**Authors:** Jigme Dorji, Amanda J. Chamberlain, Coralie M. Reich, Christy J. VanderJagt, Tuan V. Nguyen, Hans D. Daetwyler, Iona M. MacLeod

**Affiliations:** 1 Agriculture Victoria, AgriBio, Centre for AgriBioscience, Bundoora, VIC 3083 Australia; 2 Agriculture and Food, CSIRO, St Lucia, QLD 4067 Australia; 3 Global Genomics and Breeding Design Vegetable R&D, Bayer Crop Science, Bergschenhoek, The Netherlands; 4 School of Applied Systems Biology, La Trobe University, Bundoora, VIC 3083 Australia

## Abstract

**Background:**

Mitochondrial genomes differ from the nuclear genome and in humans it is known that mitochondrial variants contribute to genetic disorders. Prior to genomics, some livestock studies assessed the role of the mitochondrial genome but these were limited and inconclusive. Modern genome sequencing provides an opportunity to re-evaluate the potential impact of mitochondrial variation on livestock traits. This study first evaluated the empirical accuracy of mitochondrial sequence imputation and then used real and imputed mitochondrial sequence genotypes to study the role of mitochondrial variants on milk production traits of dairy cattle.

**Results:**

The empirical accuracy of imputation from Single Nucleotide Polymorphism (SNP) panels to mitochondrial sequence genotypes was assessed in 516 test animals of Holstein, Jersey and Red breeds using Beagle software and a sequence reference of 1883 animals. The overall accuracy estimated as the Pearson’s correlation squared (R^2^) between all imputed and real genotypes across all animals was 0.454. The low accuracy was attributed partly to the majority of variants having low minor allele frequency (MAF < 0.005) but also due to variants in the hypervariable D-loop region showing poor imputation accuracy. Beagle software provides an internal estimate of imputation accuracy (DR2), and 10 percent of the total 1927 imputed positions showed DR2 greater than 0.9 (N = 201). There were 151 sites with empirical R^2^ > 0.9 (of 954 variants segregating in the test animals) and 138 of these overlapped the sites with DR2 > 0.9. This suggests that the DR2 statistic is a reasonable proxy to select sites that are imputed with higher accuracy for downstream analyses. Accordingly, in the second part of the study mitochondrial sequence variants were imputed from real mitochondrial SNP panel genotypes of 9515 Australian Holstein, Jersey and Red dairy cattle. Then, using only sites with DR2 > 0.900 and real genotypes, we undertook a genome-wide association study (GWAS) for milk, fat and protein yields. The GWAS mitochondrial SNP effects were not significant.

**Conclusion:**

The accuracy of imputation of mitochondrial genotypes from the SNP panel to sequence was generally low. The Beagle DR2 statistic enabled selection of sites imputed with higher empirical accuracy. We recommend building larger reference populations with mitochondrial sequence to improve the accuracy of imputing less common variants and ensuring that SNP panels include common variants in the D-loop region.

**Supplementary Information:**

The online version contains supplementary material available at 10.1186/s12711-024-00931-5.

## Background

Mitochondria are the primary site for the cellular energy metabolism in eukaryotes. These organelles contain small mitochondrial (MT) genomes (~ 16 kb) that are circular, haploid, non-recombining, and are maternally inherited [1–3]. Thus within a single cell, there are multiple copies (up to thousands) of MT genomes present depending on cell types [4, 5]. This genome codes for 13 proteins which are not present in the nuclear genome, but overall these constitute less than 1% of all MT proteins (i.e., proteins in the mitochondrial compartment including those involved in metabolic and maintenance processes in the mitochondria). While many MT proteins continue to be identified and characterized, of those that are known and encoded by the nuclear genome, it is estimated that 15 percent are involved in energy metabolism and up to 25 percent in maintenance and regulation of the MT genome indicating a close inter-dependence between the two genomes [6–8]. Mutations in MT protein genes from both the MT and nuclear genomes have been implicated in mitochondrial diseases in humans [9, 10]. Therefore, it is possible that these mutations in livestock species (e.g., dairy cattle) may manifest as either detrimental or beneficial to traits that are associated with MT function (i.e., energy metabolism and utilization) in particular complex traits such as energy balance, feed efficiency and milk production [11].

Studies have shown that the MT genome of cattle is highly diverse and indicates a population structure different from that of the nuclear genome due to the maternal inheritance [12, 13]. It has been speculated that there may be as yet unaccounted for MT genetic variation that may augment the heritability and potentially improve genomic prediction accuracies for complex traits [14–17]. In fact, the MT genome has long been of interest to animal breeders. There have been attempts to understand the role and quantify the effects of MT DNA in animal production, mainly through association of maternal lines (often derived from pedigree), as well as relatively small-scale studies testing the association of several MT markers with production phenotypes [18–21].

In recent years, there has been renewed interest in studying the role of the MT genome on adaptation and production traits in livestock: investigations have included use of cybrids (cells with nuclear DNA from one source and MT genomes from a different source) [22–24], MT copy number, gene expression, mutations, haplotypes and haplogroups [25–28]. Further, the rapid developments in genomics over the last decade now enables larger scale genomic studies through the availability of whole genome sequences and computing capability [29]. In particular, whole genome sequencing of relatively large reference populations (e.g., 1000 Bull Genomes Project [30]) has enabled the recovery of MT genome sequences for large numbers of animals [12]. This provides a set of reference MT genomes that, coupled with the application of imputation, has the potential to deliver imputed whole genome MT sequence into many thousands of individuals that have appropriate MT Single Nucleotide Polymorphism (SNP) panel genotypes [27]. In turn, this would enable large-scale genome wide association studies (GWAS) involving all MT sequence variants being tested for associations with recorded traits, as well as evaluation of the proportion of genetic variance explained by the MT genome.

To date, to the best of our knowledge the imputation of whole genome sequence MT genotypes in cattle and their evaluation for association with complex traits has not previously been undertaken. Therefore, the aims of our study were the following:

- Evaluate the empirical accuracy of imputing mitochondrial sequence variants from a set of mitochondrial markers genotyped on a custom Illumina XT-50k SNP panel.
- Undertake a genome wide association study (GWAS) of real XT-50k mitochondrial SNP and imputed mitochondrial sequence variants with milk production traits.

## Methods

### Evaluating empirical accuracy of mitochondrial sequence imputation

#### Imputation reference mitochondrial sequence genotypes

In a previous study, we developed a high quality set of whole genome mitochondrial sequences of 1883 animals from the 1000 Bull Genomes project and empirically demonstrated accurate imputation of sporadic missing MT sequence variants [12]. The same MT sequence dataset consisting of more than 185 breeds and crossbreds was used as an imputation reference in this study. There was a small proportion of 'heterozygous' genotypes in the reference sequences even though the genome is haploid. This phenomenon is thought to be due to both mutant and wild-type versions of the mitochondrial genome co-existing in the sampled tissue and is referred to as ‘heteroplasmy’ [31]. However, heteroplasmy may also in part be due to mis-alignment of small segments of MT sequence that through the course of evolution have been incorporated in the nuclear DNA known as nuclear mitochondrial DNA segments (NUMTs) [32, 33]. Thus, heteroplasmic genotypes were converted to homoplasmic genotypes by assigning the base with the higher allelic read depth (among the reference and alternate alleles). We did this because previously we have observed poor empirical imputation accuracy of the MT heteroplasmic genotypes [12]. The allelic read depths of heteroplasmic genotypes were rarely equal, but in this case the genotypes were set to missing and then imputed as sporadic missing sequence genotypes following the approach described previously [12]. There was a total of 1949 segregating sites in the set of 1883 reference animals (with an average distance of 8 bp between sites given the mitochondrial genome size of 16.4 kb). First, these sequence genotypes were used as the imputation reference dataset to test the empirical accuracy of imputation. Second, these sequence genotypes were used as a reference for the imputation of real MT markers from a custom array (XT-50k) genotyped in 9515 dairy cattle.

#### Test animals for empirical evaluation of imputation accuracy

A subset of 516 animals from three dairy breeds were selected as the test individuals from the above MT sequence reference dataset for determining empirical accuracy of imputation from MT marker genotypes to MT sequence. The test dataset consisted of MT sequence genotypes from 267 Holsteins, 27 Jersey and 222 Norwegian Reds. These breeds were of interest because they were available in a larger dataset of animals used in this study for a GWAS of imputed and real MT SNP.

To test the empirical accuracy of imputation from lower density SNP marker panels to MT sequence, we used two marker panels: a custom XT-50k SNP panel and the Illumina BovineHD Beadchip panel. The custom XT-50k SNP panel included 27 MT SNP markers, all of which were a subset of the 343 MT markers on the BovineHD panel (see Additional file 1: Table S1 and Table S2). The positions of MT SNPs on the XT-50k and HD Illumina manifests were based on an older MT reference genome (AY526085.1: 16,338 bp long). Therefore, following Dorji et al. [12], they were lifted over to the newer MT reference genome (CM008198.1: 16,340 bp long) that was used for the alignment of the above reference sequences [12]. Twenty-two out of the 27 SNPs on the XT-50k and 70 of the 343 SNPs on the HD panels overlapped the polymorphic sites of the 1883 animals with reference MT sequence genotypes described above (see Additional file 1: Table S1 and S2 for the MT positions of all overlapping sites). The number of those sites segregating in the test set of 516 animals was 14 (XT-50k), 41 (HD) and 968 sequence variants (including those on SNP panels). The number of full mitochondrial haplotypes among the test animals were 16, 56 and 412 for the XT-50k, HD and sequence respectively compared to 37, 161 and 1380 in the full reference dataset. The average distances between the MT SNPs were 710 bp for the XT-50k set and 232 bp for the HD set.

#### Testing empirical accuracy of imputation to mitochondrial sequence

To maximize the size of the reference population for imputation we adopted a “leave-one-out” approach to test imputation accuracy (i.e., repeating the imputation process 516 times for each of the test animals one at a time). The MT sequence genotypes of the ‘left-out’ test animal were masked down to the MT XT-50k SNPs while the remaining 1882 animals were masked to the MT HD SNPs for use as an HD imputation reference for the left-out test individual. Similarly, the full set of sequence variants for these same 1882 animals became the sequence reference to impute the ‘left out’ test animal. Thus, the XT-50k MT genotypes of each test animal were first imputed to the HD MT SNP (a total of 48 imputed SNP = 70–22) and then to sequence (1879 imputed SNP = 1949–70) using Beagle 5.2 [34, 35]. Given the higher mutation rate of mitochondrial DNA compared to the nuclear DNA, the mixed breed population, and expectation of a large effective population size *(Ne*) for the MT genome due to maternal inheritance [36], we used the default *Ne* in Beagle. Furthermore, we tested the use of a lower *Ne* of 1000 and found no marked difference in the imputation accuracy.

Two measures of imputation accuracy were considered:

1. Pearson’s correlation squared between the original and imputed genotypes (R^2^) for each site across all animals.
2. The Beagle software provides an internal estimate of imputation accuracy (DR2) which is the squared correlation between the imputed allele dosage and the posterior probability of the unobserved true genotype [37]. Since DR2 is not estimated when using the leave-one-out approach, we obtained DR2 for each variant by imputing all the test animals together from mitochondrial XT-50k genotypes to HD and then to sequence.

### Mitochondrial sequence GWAS

#### Genotypes

A set of 13,999 cows (including Holstein, Jersey, Australian Red and crossbreds) were genotyped on a custom XT-50k SNP panel which included 27 MT SNPs as described earlier. The panel also included 45,709 markers from the nuclear genome. A quality check of the MT genotypes was applied on both SNP marker and individual animal levels. The MT SNPs with genotype call (GC) scores of < 0.5 were set to missing and SNPs failing GC score and/or missing in > 10% of animals were removed, resulting in 13 MT markers (XT-50k_Mt_) that passed this filter. Additionally, 238 animals were removed because they were missing > 10% of these MT SNP genotypes (i.e., 2 or more SNPs) leaving a total of 13,761 animals. The XT-50k nuclear marker genotypes used in our analyses also underwent similar quality control except that the GC score threshold was 0.6. This slightly higher GC score was used for the nuclear markers because these animal genotypes were previously processed to generate a high-quality XT-50k imputation reference population. There were 36,557 nuclear markers in this final set of genotypes. Any sporadic missing genotypes that remained in the nuclear XT-50k data (at less than 10%) were imputed with default settings in FImpute with no pedigree [38]. Finally, after checking for animals with near duplicate genotypes (< 200 differences out of 36,557 markers) a further 10 animals were removed, and 13,751 animals remained in the final XT-50k genotype data set.

#### Phenotypes for milk traits

Of the 13,751 cows in the final XT-50k SNP genotype set, there were 10,290 cows with records for three milk production traits: milk, fat and protein yields. These phenotypes were de-regressed proofs (corrected for herd, year, season and lactation) using records available across multiple lactations and were prepared by DataGene, the Australian national dairy evaluation organization (https://datagene.com.au). To check the accuracy of pedigree breed assignment, we undertook a principal components (PC) analysis using the genomic relationship of the nuclear XT-50k genotypes where the three breeds separated clearly on PC1 and PC2. A relatively small number of mixed crossbreds from the three breeds (N = 775) were removed, leaving a total of 9515 animals for further analysis consisting of Holstein (N = 5806), Jersey (N = 1984) and Australian Reds (N = 1725).

#### Imputation of real mitochondrial XT-50k genotypes

The XT-50k_Mt_ SNP genotypes of the 9515 animals with phenotypes were converted to VCF format using Plink 2.0 [39] with care to convert the genotypes to match the sequence reference allele format. Next, the XT-50k_Mt_ genotypes of 9515 animals were imputed to HD genotypes and then to sequence following the same approach and imputation reference set as described above (1883 animals) using Beagle 5.2 with default settings. After imputation, we applied a threshold on the Beagle software’s estimate of imputation accuracy, DR2 > 0.9, to select the most accurately imputed sites for further analyses.

#### Genome wide association studies (GWAS)

The imputed MT sequence genotypes (coded as 0 or 1 to reflect the haploid A or B genotypes) were used for single-trait multibreed GWAS of milk, fat and protein yield using a mixed linear model fitted with the GCTA software *“mlma”* option [40, 41] in the following model:

1 $$\mathbf{y}=\boldsymbol{1}\upmu +\mathbf{X}\mathbf{b}+\mathbf{w}\text{a}+\mathbf{Z}\mathbf{u}+\mathbf{e},$$

where **y** is a vector of deregressed proofs of milk, fat and protein yields, $\boldsymbol{1}$ is a vector of ones, $\upmu$ is an overall mean, **X** is a design matrix relating animal phenotypes to the fixed effects, **b** is the vector of the fixed effect of breed, **w** is the vector of animal genotypes for MT SNP *i*, coded as 0 or 1 (representing A or B genotypes) and $\text{a}$ is the fixed effect of SNP *i*, **Z** is the incidence matrix, **u** is the vector of genomic breeding values distributed as *N*(0, **G** ${{\varvec{\upsigma}}}_{\mathbf{g}}^{2}$) where ${{\varvec{\upsigma}}}_{\mathbf{g}}^{2}$ is the additive genetic variance, **G** is the genomic relationship matrix generated from the nuclear XT-50k genotypes and **e** is the vector of residual effects distributed as N(0, ${{\varvec{\upsigma}}}_{\mathbf{e}}^{2}$) where ${{\varvec{\upsigma}}}_{\mathbf{e}}^{2}$ is the error variance. The breed was included as a fixed effect because the production records were not previously corrected for breed.

The SNP effects were tested for significance using a stringent Bonferroni corrected p-value, defined as 0.01/N, where N = number of SNPs in the MT test set that were not in perfect LD.

## Results

### Empirical accuracy of imputation of mitochondrial XT-50k to sequence

The MT sites on XT-50k of 516 test animals were first imputed to HD markers and then to MT whole genome sequence variants. Of the 48 imputed HD MT SNP, only 19 had a MAF > 0.005 in the reference genotypes, and 353 of the 1,879 imputed sequence variants had a MAF > 0.005 (see Additional file 1: Table S3). There were 63 imputed variants with MAF > 0.01 and just 11 variants with a MAF > 0.1. The overall accuracy of imputation measured as Pearson’s correlation (R) between original and imputed genotypes coded as 0 (i.e., major allele genotype) and 1 (minor allele genotype) across all imputed sites and all test animals was 0.409 (R^2^ = 0.167) for XT-50k to HD imputation and 0.67 (R^2^ = 0.449) for XT-50k to sequence via HD. The accuracy of imputation for HD variants was impacted by having a higher proportion of D-loop variants with MAF > 0.02 in the test animals (18.5% compared to the 2.7% in the sequence variants) and the imputation accuracy for these higher frequency D-loop variants was very poor. We tested the imputation accuracy of imputing direct to sequence from the XT-50k variants but this did not improve the imputation accuracy for the HD variants. The average R^2^ per site, calculated for 240 sites that segregated in both the genotyped and imputed test animal data, was 0.842 (0.777 for HD sites and 0.848 for sequence) and 151 had an R^2^ > 0.9. Of these 240 sites, 233 had a MAF > 0.005 and showed an average R^2^ = 0.844 and 147 of these sites were imputed with very high accuracy of R^2^ > 0.9 (see Additional file 1: Table S3). As expected, there was a bias towards the minor allele being wrongly imputed to the major allele, and in our data the major allele always represented the reference allele except for two sites in the D-loop region (at 169 and 364 bp). Thus, when considering all sites segregating in the test animals and with MAF < 0.005 in the reference set, the average error rate for the imputed alternate allele was 98.9% indicating the difficulty of accurate imputation for less common alleles.

The Beagle software provides an internal estimate of imputation accuracy (the DR2 statistic) that can be used as a means of filtering poorly imputed variants before downstream analyses and therefore it was of interest to compare the DR2 with the empirical accuracy measures. The DR2 was obtained by imputing the entire test dataset altogether from their XT-50k genotypes to sequence via HD genotypes because the leave one out approach does not produce DR2 estimates. Thus, this DR2 may somewhat underestimate the leave-one-out imputation accuracy. Nonetheless, 10% of the imputed variants (201 out of 1927 imputed HD & sequence sites) had DR2 > 0.9 (mean 0.996, min 0.920, max 1.0) and they overlapped with 138 sites of the 151 sites with an empirical R^2^ > 0.9. The remaining 58 variants with an estimable R^2^ < 0.9 and DR2 > 0.9 showed an average R^2^ of 0.626 (min 0.112 and max 0.831) and 31 variants of the 58 had R^2^ > 0.7 (see Additional file 1: Table S3). This indicates that filtering on a DR2 threshold is useful to identify the more accurately imputed variants for further analyses as found in other studies [42, 43].

A more detailed check on the imputation errors per site showed that they were spread over 42% of the imputed sequence positions considered (i.e., 805 out of 1927 imputed sites) but increased to 83.6% when considering only the sites segregating in the real sequence of the test animals. Of those sequence positions showing imputation errors, the majority (73%) had only 1 to 2 errors (across 516 animals) with the exception of four sequence variants (at positions 169, 215, 364, 1595, 16,318 bp) that had extremely low accuracies (R^2^ ranging from 0.000 to 0.049: see Additional file 1: Table S3). The genotypes in these positions were not specific to any particular breed and all but one (at 1595 bp) site are located within the D-loop region of the MT genome. These four D-loop variants together with others in the D-loop contributed almost 50% of the wrongly imputed genotypes while only 10% of the imputed variants fall in this region (198 out of 1927). Thus the D-loop was the most poorly imputed region. On a per animal basis, the vast majority of animals (90%) had less than 10 errors (< 0.6%) while the overall range was 0 to 18 errors (< 1%).

### Genome-wide association study using mitochondrial SNPs

For this second part of the study, we used 9515 cows with imputed MT sequence genotypes and custom XT-50k SNP array genotypes (nuclear genome) as well as milk, fat and protein yield phenotypes. The MT sequence genotypes were imputed from real genotypes on the XT-50k panel and it is likely that these were a more accurate starting set of genotypes than those used for our empirical study (the latter being masked-down sequence data). Additionally the entire MT sequence reference population was used for the imputation of the 9515 cows and our empirical test of imputation accuracy demonstrated that the Beagle DR2 is a useful indicator for filtering poorly imputed variants. Therefore here, we applied a Beagle DR2 > 0.9 from the imputation of the 9515 animals as a threshold to keep only 216 imputed sequence variants. In addition there were 13 directly genotyped MT markers from the XT-50k custom SNP panel making a total of 229 variants. We did not filter on empirical R^2^ from the first part of the study because not all variants segregating in this set of animals were segregating in the test animal set (thus previously had no estimable R^2^). Furthermore, the starting set of real genotypes showed a better spread of MAF which may have resulted in different imputation accuracy to that of the 516 test animals. Nonetheless, of the 216 variants with DR2 > 0.9, we found 207 variants had an empirical R^2^ estimate: of these 175 (85%) had R^2^ > 0.7 and 161 had R^2^ > 0.8.

The GWAS tested the effect of each of the 227 MT variants. In this set of 9915 animals, many pairs of the imputed MT variants were in close to perfect LD (r^2^ > 0.9), such that if one of each pair was pruned out this would leave only 18 variants. Interestingly most of the variant pairs that were in perfect LD had a MAF of 0.3807 including four of the XT-50k real genotypes, and the others ranging between 0.0002 and 0.3872. We did not prune the variants a priori for the GWAS because we wanted to test for putative causal variants. However, the estimated MT variant effects across breeds for milk, fat and protein yields were not significant at p < 0.01 (applying a Bonferroni correction for 18 independent tests) (see Additional file 1: Table S4).

## Discussion

To our knowledge, this is the first study in cattle to investigate the empirical accuracy of imputation from a SNP panel to whole MT genomes, and to evaluate the effect of real and imputed MT genotypes on milk yield traits in more than 9000 dairy cattle. The present study provides useful insights for future studies in this area, and this is a key focus of our discussion.

In an earlier study [12] the high accuracy of imputing sporadic missing MT sequence genotypes suggested the potential for exploiting existing data for population scale imputation of MT genomes from lower density SNP. However, in the present study the empirical accuracy of imputed sequence sites from SNP panel genotypes was rather variable and there are some important lessons learnt from this study. First, the accuracy of imputation tended to be lower for variants in the D-loop, a short non-coding region on the MT genome, compared to the coding region. This is perhaps not surprising because the D-loop region is known to be more highly variable (potentially a mutational hotspot) than the remaining coding region, showing greater diversity both between and within breed [12, 44–46] and thus is more likely to be poorly imputed. Of the 12 variants with MAF > 0.1, 11 were in the D-loop, while the MAF of the XT-50k MT SNP were all < 0.02 in the test and reference animals. Therefore, it appears that the XT-50k MT SNP were unable to closely tag the common D-loop SNPs, resulting in the low imputation accuracy for this region. Unfortunately, only one variant on the XT-50k panel was from the D-loop region, therefore, in future studies it may be advisable to include some of the more common D-loop SNPs on panel designs. Additionally, it is difficult to impute rare variants accurately and only 372 (20%) of the imputed SNP had a MAF > 0.005 and only 3% had a MAF > 0.01 in the reference population. Therefore, it would be advisable to increase the size of the imputation reference population by several fold and apply a threshold on the minor allele count of variants used for imputation. Further, it is likely that increased MT marker density on SNP arrays for those SNP that segregate at sequence level with a range of MAF (including in the D-loop region) would provide further improvements in imputation accuracy of the alternate alleles. A recent study in humans, with a reference population of almost 40,000 MT genomes, showed that the imputation accuracy for MT sequence variants was generally higher for denser MT SNP panels and for panels that included MT variants with a range of MAF [46]. Interestingly, the MAF of variants in the real XT-50k genotypes of 9515 cows showed more variation than observed in the 516 test animals and ranged from 0.00084 to 0.3872. This is perhaps expected given that the animals used for our empirical evaluation of imputation accuracy were bulls that were included in the 1000 Bull Genomes Project because they were highly influential ancestors of specific dairy cattle populations and many may have shared the same maternal lines. However, the set of 9515 cows used for the GWAS were from a range of commercial herds across Australia potentially representing different proportions of haplogroups than found in the 1000 Bull Genome Project set.

A weakness of our evaluation of empirical accuracy is that the existing sequence genotype calls were assumed to be always correct, while it is known that there will be sequencing and alignment errors. In particular, the short-read sequence data used here may include alignment errors due to small regions of MT sequence that are also found with high similarity in nuclear DNA due to transfer events across evolutionary time (often referred to as “NUMTs”). Once these NUMTs become part of the nuclear genome they may undergo mutational events and if misaligned to the MT genome can result in false positive segregating MT SNPs [47, 48]. This can potentially give rise to erroneous heteroplasmy, where an individual is found to have some heterozygous MT sites even though the genome is haploid. A complication of dealing with heteroplasmy is that it is also possible (though not common) for this to arise naturally through mutations of the MT genome, occasional leakage of paternal MT DNA and through inheritance from a heteroplasmic egg itself [33]. Therefore, when we previously generated our imputation reference population, we had imposed a read depth filter to identify and exclude sites that might be contaminated by NUMT alleles [12]. In a previous study, we found that imputation of sporadic missing genotypes at masked heteroplasmic sites showed low imputation accuracy [12] and therefore for the few remaining sites with heteroplasmy in this study, we assigned the most common allele to be the genotype. Ideally in the future, reference populations of MT sequences could be developed using long-read sequencing technology where the entire, or almost entire length of the MT genome will be sequenced in a single read. While previously the long-read technology was plagued by low base call accuracy, this has now improved to the level of short-read technology [49–51]. Thus, the long-read approach should now help to resolve the negative influence of NUMTs and the question of heteroplasmy.

It is plausible to speculate that MT variants might affect milk trait phenotypes because of the high energy metabolism requirements for milk production (reviewed in [52]). In addition, a previous study reported association of MT SNP (e.g., variant at 169 bp which is in the D-loop and was poorly imputed in our study) with milk traits [20]. Our finding of no significant variants for milk trait GWAS in this study is however somewhat consistent with a study [17] which reported cytoplasmic and maternal inheritance was negligible [53]. Outside of the small D loop region, the MT genome is mainly comprised of coding regions (with no introns) and the MT genes are generally found to be highly co-expressed [54] because their transcription is controlled by a single regulatory region and transcribed as a single unit [3]. Therefore compared to the nuclear genome, it is much more likely that a mutation in the MT genome will affect coding sequence and create a missense variant. However, the vast majority of proteins required for MT function are encoded by the nuclear genome: thus mutations in coding or regulatory regions of these genes on the nuclear genome are potentially more likely to affect energy demanding traits such as milk production. In a recent study of MT protein gene expression in high and low feed efficient dairy cattle (another trait related to energy utilization) [11], there was enrichment of nuclear encoded MT protein genes among those differentially expressed but no enrichment for genes from the MT genome. Therefore, the finding of no significant effects of MT sequence variants in our GWAS may be a true reflection of these variants tending to have small to negligible genetic influence on the traits. It is also possible that the power of GWAS may have been considerably reduced as a result of imputation errors because based on our empirical test of imputation accuracy, we know that alternate allele MT genotypes were imputed with lower accuracy compared to reference alleles. However, at least some of the variants tested were directly genotyped on the XT-50k panel. Additionally, given the range in MAF of the real XT-50k MT genotypes it might be expected that some at least would tag any underlying moderate size MT causal variants for the milk traits if present.

To some extent these studies question the likelihood that variants in the MT genome will have a strong influence on traits of economic importance in dairy cattle. It seems plausible that MT mutations with a significantly unfavorable impact on milk production or other key traits are under strong negative selection because a cow with such a mutation may be culled from the herd or not used to breed replacement heifers. Thus, only unfavorable mutations with small effects would remain in the breeding population and as such only a very limited number of mutations are found in the small MT genome compared to the nuclear genome. Additionally, even newly arising favorable MT mutations would tend to remain at low allele frequency because they must be inherited through the maternal line, versus favorable nuclear DNA mutations that can be rapidly disseminated into the population through use of artificial insemination.

## Conclusions

In conclusion, with the available reference population we found that the imputation accuracy for mitochondrial sequence genotypes from a 50k SNP array was low for the majority of variants. The low minor allele frequency of many mitochondrial SNPs combined with the hyper-variability of the D-loop region indicate that a much larger reference population is needed for the accurate imputation of MT sequence variants. It is also advisable to design genotyping platforms that capture relatively dense coverage of MT variants at a range of MAF. The GWAS study here may have lacked power due to imputation errors, but may also suggest that any existing MT effects for milk traits are rare and/or small.

## Supplementary Information

Additional file 1: Table S1. XT-50k mitochondrial genotyping positions, SNP name and lift over position on the mitochondrial reference genome CM008198.1. Table S2. List of mitochondrial HD SNP markers overlapping reference population genotypes. Table S3. Summary statistics of imputation accuracies for mitochondrial sequence variants in the reference and validation population. Table S4. WAS results for milk traits using 9515 cows with real mitochondrial xt-50k genotypes and imputed sequence variants (DR2 > 0.9).

## Data Availability

The mitochondrial sequences for part of the mitochondrial reference set used in this study was previously made available (https://doi.org/10.1038/s41598-022-09427-y). DataGene Limited (http://www.datagene.com.au/) manages the phenotype and genotype data of Australian dairy animals and access to these data for research purposes may be granted upon written request to DataGene. Requests for research access to the MT data may be made by contacting the senior author at Agriculture Victoria, Australia.
